# Tissue-Specific Expression Patterns of MicroRNA during Acute Graft-versus-Host Disease in the Rat

**DOI:** 10.3389/fimmu.2016.00361

**Published:** 2016-09-16

**Authors:** Dasaradha Jalapothu, Margherita Boieri, Rachel E. Crossland, Pranali Shah, Isha A. Butt, Jean Norden, Ralf Dressel, Anne M. Dickinson, Marit Inngjerdingen

**Affiliations:** ^1^Department of Molecular Medicine, Institute of Basic Medical Sciences, University of Oslo, Oslo, Norway; ^2^Department of Immunology, Oslo University Hospital – Rikshospitalet, Oslo, Norway; ^3^Institute of Cellular Medicine, Medical School, Newcastle University, Newcastle-upon-Tyne, UK; ^4^Institute of Cellular and Molecular Immunology, University Medical Center Göttingen, Göttingen, Germany

**Keywords:** aGvHD, T cells, skin, miRNA, gut

## Abstract

MicroRNAs (miRNA) have emerged as central regulators of diverse biological processes and contribute to driving pathology in several diseases. Acute graft-versus-host disease (aGvHD) represents a major complication after allogeneic hematopoietic stem cell transplantation, caused by alloreactive donor T cells attacking host tissues leading to inflammation and tissue destruction. Changes in miRNA expression patterns occur during aGvHD, and we hypothesized that we could identify miRNA signatures in target tissues of aGvHD that may potentially help understand the underlying molecular pathology of the disease. We utilized a rat model of aGvHD with transplantation of fully MHC-mismatched T cell depleted bone marrow, followed by infusion of donor T cells. The expression pattern of 423 rat miRNAs was investigated in skin, gut, and lung tissues and intestinal T cells with the NanoString hybridization platform, in combination with validation by quantitative PCR. MHC-matched transplanted rats were included as controls. In the skin, upregulation of miR-34b and downregulation of miR-326 was observed, while in the intestines, we detected downregulation of miR-743b and a trend toward downregulation of miR-345-5p. Thus, tissue-specific expression patterns of miRNAs were observed. Neither miR-326 nor miR-743b has previously been associated with aGvHD. Moreover, we identified upregulation of miR-146a and miR-155 in skin tissue of rats suffering from aGvHD. Analysis of intestinal T cells indicated 23 miRNAs differentially regulated between aGvHD and controls. Two of these miRNAs were differentially expressed either in skin (miR-326) or in intestinal (miR-345-5p) tissue. Comparison of intestinal and peripheral blood T cells indicated common dysregulated expression of miR-99a, miR-223, miR-326, and miR-345-5p. Analysis of predicted gene targets for these miRNAs indicated potential targeting of an inflammatory network both in skin and in the intestines that may further regulate inflammatory cytokine production. In conclusion, comprehensive miRNA profiling in rats suffering from aGvHD demonstrate tissue-specific differences in the expression patterns of miRNA that may not be detected by profiling of peripheral blood T cells alone. These tissue-specific miRNAs may contribute to distinct pathologic mechanisms and could represent potential targets for therapy.

## Introduction

Allogeneic hematopoietic stem cell transplantation (allo-HSCT) is a treatment option for hematological malignancies or autoimmune disorders. Acute graft-versus-host disease (aGvHD) represents a major complication after allo-HSCT with high morbidity and mortality, next to other complications, such as relapse of the malignancy, engraftment failure, or opportunistic infections (1). GvHD is evoked by donor T cells present in the graft that recognize and attack mismatched host tissues in an immunosuppressed environment. Host antigen-presenting cells (APC) are activated as a consequence of damage to epithelial barriers and the ensuing inflammation that is triggered by the pretransplant conditioning. Donor T cells are activated by host APC in draining lymph nodes, enter the circulation, and extravasate into the gastrointestinal tract, skin, lung, and liver, representing major target tissues for aGvHD, where they mediate further increased inflammation and tissue damage (2).

Recent years have seen a surge of interest in the potential of microRNAs (miRNAs) as both biomarkers to predict GvHD development and targets for therapeutic interventions (3–6). miRNAs are small non-coding RNAs of 19–22 nucleotides in length that negatively influence protein expression by either degrading messenger RNA (mRNA) or repressing its translation by binding to the 3'-untranslated region (7). miRNAs are potent regulators of biological processes and have been shown to play central roles in the regulation of immune responses (8). miRNAs only partially pair with their target mRNA, implying that a single miRNA can target several different mRNA molecules and a given mRNA can be targeted by several miRNAs (9). This infers that miRNAs are part of a complex regulatory network, potentially involving several different miRNAs simultaneously. Several miRNAs have been identified as critical regulators of T-cell activity and hence have been associated with several cancers, autoimmune diseases, and GvHD (5). Particular miRNAs associated with aGvHD include miRNAs that enhance T-cell activation, such as miR-155 (4, 10), miR-142 (11), miR-29a, miR-29b, and the miR-17-92 cluster (12), and miRNAs that repress T-cell activation, such as mir-146a (13), which is also upregulated in T regulatory cells (Tregs) (14).

The expression patterns of miRNA change in response to external factors, and their expression patterns can be tissue and cell specific (15, 16). As aGvHD affects several distinct organs, we hypothesized that discrete miRNA signatures could be identified in different target tissues of aGvHD that may potentially help understand the underlying molecular pathology of the disease. We undertook a study to comprehensively analyze and compare the miRNA expression signature in three classical target tissues for aGvHD: skin, intestines, and lung. For this purpose, we obtained gut, skin, and lung tissues from rats suffering from aGvHD, as well as from T cells isolated from the intestines or peripheral blood and analyzed miRNA expression using the NanoString platform and quantitative real-time PCR (qRT-PCR). This study thus represents a systematic approach for profiling of differentially expressed miRNA in target tissues during aGvHD. We also identified a regulatory network of miRNAs and their predicted gene targets, consisting mainly of cytokines and their transcriptional regulators.

## Materials and Methods

### Animals and Ethical Considerations

Male rats aged 8- to 12-weeks of the strains PVG (*RT1^c^*, CD45.1), PVG.7B (*RT1^c^*, CD45.2), and the MHC congenic rat strain PVG.1N (*RT1^n^*, CD45.1) were used. The PVG.1N rat strain has the same genetic background as the PVG strain with the exception of the MHC complex that is from the Brown Norway rat. The rats have been maintained at the Department of Comparative Medicine, Institute of Basic Medical Sciences, University of Oslo, for more than 20 generations. The Department of Comparative Medicines institutional veterinarian has established the rules for feeding, monitoring, handling, and sacrifice of animals in compliance with regulations set by the Ministry of Agriculture of Norway and “The European Convention for the Protection of Vertebrate Animals used for Experimental and other Scientific Purposes.” The institutional veterinarian has delegated authority from the Norwegian Animal Research Authority (NARA). The laboratory animal facilities are subject to a routine health-monitoring program and tested for infectious organisms according to a modification of Federation of European Laboratory Animal Science Associations (FELASA) recommendations. The use of animals for this study was approved by NARA, license number 6013 (*in vitro*) and 6060 (*in vivo*). Rats were sacrificed by asphyxiation with CO_2_ in a chamber allowing controlled input of gas, such as to reduce suffering of the animals.

### Bone Marrow Transplantation and Induction of Acute GvHD

Transplantation with 3 × 10^7^ T-cell depleted bone marrow cells was performed with PVG.7B rats as donors and age-matched MHC-mismatched PVG.1N (*n* = 6) or MHC-matched PVG rats (*n* = 6) as recipients after irradiation at 8.5 Gy from a ^137^Cs source (Gammacell 3000 Elan, MDS Nordion, USA) under anesthesia with ZRF cocktail (3.3 mg Zolazepam, 3.3 mg Tiletamine, 0.5 mg Xylazine, and 2.6 μg Fentanyl per ml 0.9% NaCl; 1 ml/kg). Bone marrow was obtained by flushing the tibias and femurs with RPMI containing 2% FBS, followed by passage through 70 μm nylon cell strainer (BD Biosciences), and separation of mononuclear cells by density gradient centrifugation on Lymphoprep™ 1.077A (Axis-Shield, Oslo, Norway). T cells were depleted with a combination of anti-CD5 (OX19, mIgG1) and anti-TCRαβ (R73, mIgG1) antibodies and anti-mouse IgG coated Dynabeads (Thermo Fisher Scientific, Waltham, MA, USA). The number of CD3^+^ T cells was reduced more than 10-fold, from 3.0 to 0.2% as assessed by flow cytometry. To induce acute GvHD, 1 × 10^7^ cervical and mesenteric lymph node cells (~60% CD3^+^NKR-P1A^−^ T cells as evaluated by flow cytometry) from PVG.7B rats were infused into recipients 14 days post transplantation. Weight and GvHD symptoms (activity, kyphosis, skin, and fur integrity) were assessed twice weekly. Rats with severe weight loss (>15%) or evident signs of disease (severe skin lesions) were sacrificed 12–14 days after T-cell infusion. Scoring was performed non-blinded. In this model, most severe pathology is observed in the skin and oral mucosae.

### Isolation of Tissues and T Cells

Immediately after sacrifice, tissue samples of small intestines, liver, lung, and skin were dissected and stored in RNAlater^®^ (Thermo Fisher Scientific) at −80°C until further processing. Peripheral blood mononuclear cells (PBMCs) were prepared from heparinized peripheral blood by Lymphoprep™ separation (Axis-Shield, Oslo, Norway). Intraepithelial lymphocytes (IELs) from the small intestine were isolated as previously described (17). T cells were enriched from PBMCs or IELs by positive immunomagnetic selection with Dynabeads^®^ Pan Mouse IgG (Thermo Fisher Scientific) coated with anti-rat TCRαβ antibodies (clone R73, mIgG1). T cells were extensively washed in PBS, pelleted, added RNAlater^®^, and stored at −80°C until further use (>6 months).

### RNA Extraction, cDNA Synthesis, and Quantitative Real-Time PCR

Total RNA was extracted from skin, lung, intestinal, or liver tissue, or from intestinal or peripheral blood T cells isolated from control animals (*n* = 6) or aGvHD animals (*n* = 6) using the *miR*Vana miRNA Isolation Kit (Ambion, Austin, TX, USA). Tissue RNA was also isolated with TRI reagent (Ambion, Thermo Fisher Scientific), in accordance with the manufacturer’s instructions and using RNase-free equipment. For RNA assessed by NanoString, RNA concentration and RNA integrity number (RIN) were measured using the Bioanalyzer 2100 and RNA 6000 Nano Kit (Agilent Technologies), and only samples with RIN values ≥7 were included. For RNA isolated by TRI reagent, purity and concentration was measured by 260:280 and 260:230 ratios (purity measured between 1.8 and 2.0) using NanoDrop 1000 spectrophotometer (Thermo Fisher Scientific), and the integrity was assessed by gel electrophoresis.

For miRNA expression analysis, 10 ng of total RNA was reverse transcribed using the TaqMan^®^ MicroRNA Reverse Transcription Kit (Applied Biosystems) and specific TaqMan^®^ MicroRNA/Endogenous Control RT Assay for 4.5S RNA (H) A, 4.5S RNA (H) B, snoRNA, U87, miR-19b, miR-20a, miR-21, miR-22, miR-29a, miR-29b, miR-30b-5p, miR-34b, miR-99a, miR-142-5p, miR-146a, miR-155, miR-223, miR-326, miR-344a, miR-345-5p, miR-466b, miR-542-5p, miR743b, miR-874, and miR-3573. qRT-PCR was performed using the SensiFAST probe Hi-ROX kit (Bioline) and specific TaqMan^®^ MicroRNA/Endogenous Control TM Assays in a total volume of 10 μl/reaction [5 μl SensiFAST Bioline probe Hi-ROX mix, 0.5 μl Taqman (TM) assay, 1.5 μl ddH_2_O, and 3 μl (3 ng) of cDNA], and cycling conditions comprised 10 min at 95°C and then 40 cycles at 95°C (15 s) and 60°C (1 min). miRNA expression levels were normalized with respect to the endogenous controls and expression levels reported as log of 2^−ΔΔCt^.

To generate cDNA for mRNA expression studies, 1 μg of RNA was mixed with 2 μl of random hexamer primers (Promega, Madison, WI, USA) and ddH_2_O up to a volume of 15 μl, and run for 10 min at 70°C. One microliter of M-MLV reverse transcriptase, 5 μl of M-MLV buffer, 1 μl of RNAsin (all from Promega, Madison, WI, USA), 0.01M of DTT (1 μl, Sigma-Aldrich), and 2 μl of 10 mM dNTPs were then added to a total volume of 25 μl and run for 1 h at 37°C. cDNA was stored at −20°C until use. Real-time PCR was performed with Power SYBR^®^ Green PCR Master Mix (Applied Biosystem, Waltham, MA, USA) using 7900HT Fast Real-Time PCR System (Life Technologies, Waltham, MA, USA) and initially run for 2 min at 50°C and 10 min at 95°C, followed by 40 cycles of denaturation at 95°C (15 s) and amplification at 60°C (1 min). Melting curve analysis was performed at 95°C (15 s) followed by 60°C (20 s) and 95°C (20 s). Primers were designed using NCBI PrimerBlast, and specificity was tested *in silico* by BLAST or MFEprimer-2.0. Primers are listed in Table 1. mRNA expression levels were normalized with respect to β-2-microglobulin (*B2m*). *B2m* was chosen as the preferred reference gene due to its stable expression across tissues and between aGvHD and control samples compared to another tested reference gene *Gapdh*, using the algorithm NormFinder.^1^ Expression levels were reported as log of 2^−ΔΔCt^.

**Table 1:** **List of primers for mRNA expression analysis**.

Gene	Primer sequence 5′–3′	Accession ID
*B2m*	F: GAGCAGGTTGCTCCACAGGT	NM_012512.2
	R: CAAGCTTTGAGTGCAAGAGATTGA	
*Ccl5*	F: CTTGCAGTCGTCTTTGTCACTC	NM_031116.3
	R: CACTTCTTCTCTGGGTTGGCA	
*Ccl22*	F: TGCTTCAGACTTCCTTGGCC	NM_057203.1
	R: GTGCTGGAGACCGAGAAACA	
*Cxcl10*	F: TGCAAGTCTATCCTGTCCGC	NM_139089.1
	R: ACGGAGCTCTTTTTGACCTTC	
*Cxcl11*	F: CGGTTCCAGGCTTCGTTATG	NM_182952.2
	R: CTTGCTTGGATGTGGGGTCC	
*Ets1*	F: ACGACTACCCTTCCGTCATTC	NM_012555.2
	R: CGGTCACAACTATCGTAGCTCT	
*Gapdh*	F: GGGCTGCCTTCTCTTCTGAC	NM_017008.3
	R: CGCCAGTAGACTCCACGACA	
*Hdac1*	F: CTCCATCTTCTCTCCAAGTCCCT	NM_001025409.1
	R: GTGCGCTGGTCCCTATCTAGT	
*Hdac3*	F: CTGGGAGGTGGTGGTTACAC	NM_053448.1
	R: TCTGATTCTCGATGCGGGTG	
*IL10*	F: CCTCTGGATACAGCTGCGAC	NM_012854.2
	R: TAGACACCTTTGTCTTGGAGCTTA	
*IL18*	F: ACCTGAAGATAATGGAGACTTGG	NM_019165.1
	R: TCTGGTCTGGGATTCGTTGG	
*IL22*	F: TCCAGCAGCCATACATCGTC	NM_001191988.1
	R: GACTGGGGGAGCAGAACATC	
*Il6r*	F: CTATACCCCTGCCCACATTCC	NM_017020.3
	R: GACTTCCATTTCTTCTTGAGTCTC	
*Notch1*	F: TCAGCTCCCTGCAAGAATGG	NM_001105721.1
	R: ATCGATGCCTCGCTTCTGTG	
*Snai1*	F: AGCAGAGTTGTCTACCGACC	NM_053805.1
	R: GGAAGGTGAACTCCACACAC	
*Tgfb*	F: AGGGCTACCATGCCAACTTC	NM_021578.2
	R: CCACGTAGTAGACGATGGGC	
*Tgfbr2*	F: AGCTCTAACATCCTAGTAAGAAGCG	NM_031132.3
	R: GTATCTCGCTGTTCCCACCTG	

### miRNA Expression Analysis by NanoString nCounter System miRNA Assay

Expression levels of 423 different miRNAs were measured using the nCounter^®^ Rat v1.5 miRNA Expression Assay Kit from NanoString Technologies, Seattle, WA, USA. In this assay, miRNAs are detected and counted by hybridization with fluorescently labeled barcoded probes, followed by scanning and expression quantification. The assay incorporates 423 mature miRNAs based on miRBase version 17 and includes 6 positive mRNA controls, 8 negative mRNA controls, 3 ligation positive, 3 ligation negative synthetic miRNA controls, and 4 mRNA housekeeping controls (*Act6, B2m, Gapdh*, and *Rpl19*). As starting material 100 ng of RNA from T cells and 150 ng of RNA from tissues were used. Three control rats and three aGvHD rats were used for the analysis. Data normalization was performed using nSolver Analysis Software v2.5 (NanoString Technologies) incorporating positive and negative background subtraction based on geometric means and codeset content normalization to housekeeper genes established using geometric means. After normalization, one aGvHD gut sample and one control skin sample were excluded from further analysis due to quality control issues with content normalization.

### Ingenuity Pathway Analysis

To explore possible interactions between miRNAs and their putative gene targets, pathway analysis was performed using the QIAGEN Ingenuity Pathway Analysis (IPA) software (Ingenuity Systems, Redwood City, CA, USA). Pathway analysis was restricted to predicted targets, both direct and indirect, of validated dysregulated miRNAs. miRNA target prediction analyses were filtered for genes involved in T cell activation, T cell migration, T cell proliferation, and GvHD and other inflammatory pathways. Based on these inputs, a regulatory network was built around the dysregulated miRNAs in skin or gut, in order to identify potential gene targets.

### Statistical Analysis

Quantitative PCR data were analyzed using GraphPad Prism 5 (La Jolla, CA, USA). The difference between two groups was examined using the Student’s *t*-test (two-tailed) with Welch’s correction. NanoString data analysis for fold change expression differences between two groups was calculated using nSolver v2.5 (NanoString Technologies) based on normalized ratio data. Further analysis was performed using a pipeline designed by Newcastle University, Hematological Sciences Department, to integrate a number of “R” statistical packages in the “R” programming language, which are publically available *via* Bioconductor.^2^ *p* values between two groups were generated using a two-tailed *t*-test. Volcano plots were generated using functions within the “ggplot2” package, and heatmaps were constructed using “gplots” and “RColorBrewer,” based on an unsupervised clustering approach with a Euclidean distance measure and “Complete” as the agglomeration method.

## Results

### miR-326 and miR-34b Are Dysregulated in Skin and May Potentially Target Notch1 and Inflammatory Cytokine Genes

Acute GvHD was induced in rats by transplanting T-cell-depleted bone marrow from PVG.7B strain donors (*RT1c*, CD45.2 allotype) to irradiated MHC-mismatched PVG.1N strain recipients (*RT1n*, CD45.1 allotype), followed 14 days later by infusion of donor T cells from PVG.7B. As controls, irradiated PVG (*RT1c*, CD45.1 allotype) recipients were transplanted with MHC-matched PVG.7B T-cell depleted bone marrow and infused with donor T cells at day 14. No symptoms of aGvHD developed in these animals, but using the same protocol ensured that differential expression of miRNAs would specifically be due to aGvHD responses. With this protocol, rats started showing symptoms of aGvHD around day 12 after DLI and worsened rapidly as observed by rapidly progressing weight loss (Figure S1 in Supplementary Material), ruffled fur, skin lesions, and kyphosis. Rats with the strongest symptoms had severe leukopenia in the spleen. Total RNA was isolated from gut, skin, liver, and lung tissues from rats with aGvHD or from control rats. To obtain miRNA expression signatures in these target organs of aGvHD, expression of 423 rat miRNAs were tested using the nCounter^®^ NanoString hybridization platform.

**Figure 1:**
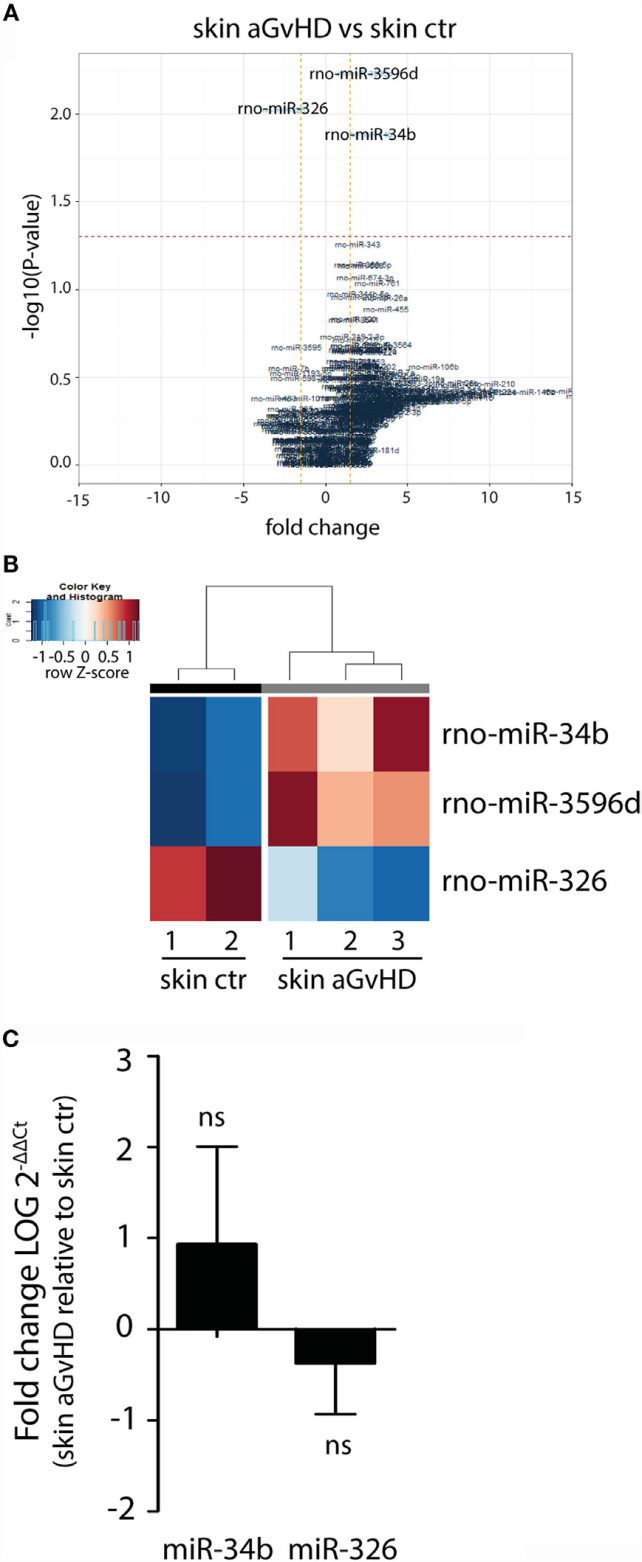
**miRNA expression signature in skin during aGvHD**. **(A)** Volcano plot showing the relationship between fold change and significance between the two groups. The horizontal dashed line indicates cutoff for significance *p* < 0.05 (−log10 *p*-value >1.3) and the vertical lines for fold change ≥1.5/≤−1.5. **(B)** Heatmap showing unsupervised hierarchical clustering of differentially expressed miRNAs in skin between control (*n* = 2) and aGvHD (*n* = 3) animals. Each column represents an individual rat. Relative fold change is indicated by the color scale (red: high; blue: low). **(C)** qRT-PCR analysis of miR-34b and miR-326 expression in skin of control (*n* = 6) or aGvHD (*n* = 6) rats. Samples were normalized to endogenous controls and expression levels reported as log of 2^−ΔΔCt^ ± SD. Significance was calculated using the Student’s *t*-test (two-tailed) with Welch’s correction.

Assessment of normalized expression data for all miRNAs in skin samples obtained from both aGvHD and control rats showed significant differential expression of three miRNAs, with upregulation of miR-34b (2.7-fold change, *p* = 0.013) and miR-3596d (2.4-fold change, *p* = 0.006) and downregulation of miR-326 (−2.6-fold change, *p* = 0.009) in aGvHD samples (Figures 1A,B). Further validation by qRT-PCR analysis of skin samples obtained from the same and another independent transplantation experiment showed trends toward increased expression of miR-34b and decreased expression of miR-326 (Figure 1C). Unavailability of a TaqMan probe against rno-mir-3596d prevented its validation.

**Figure 2:**
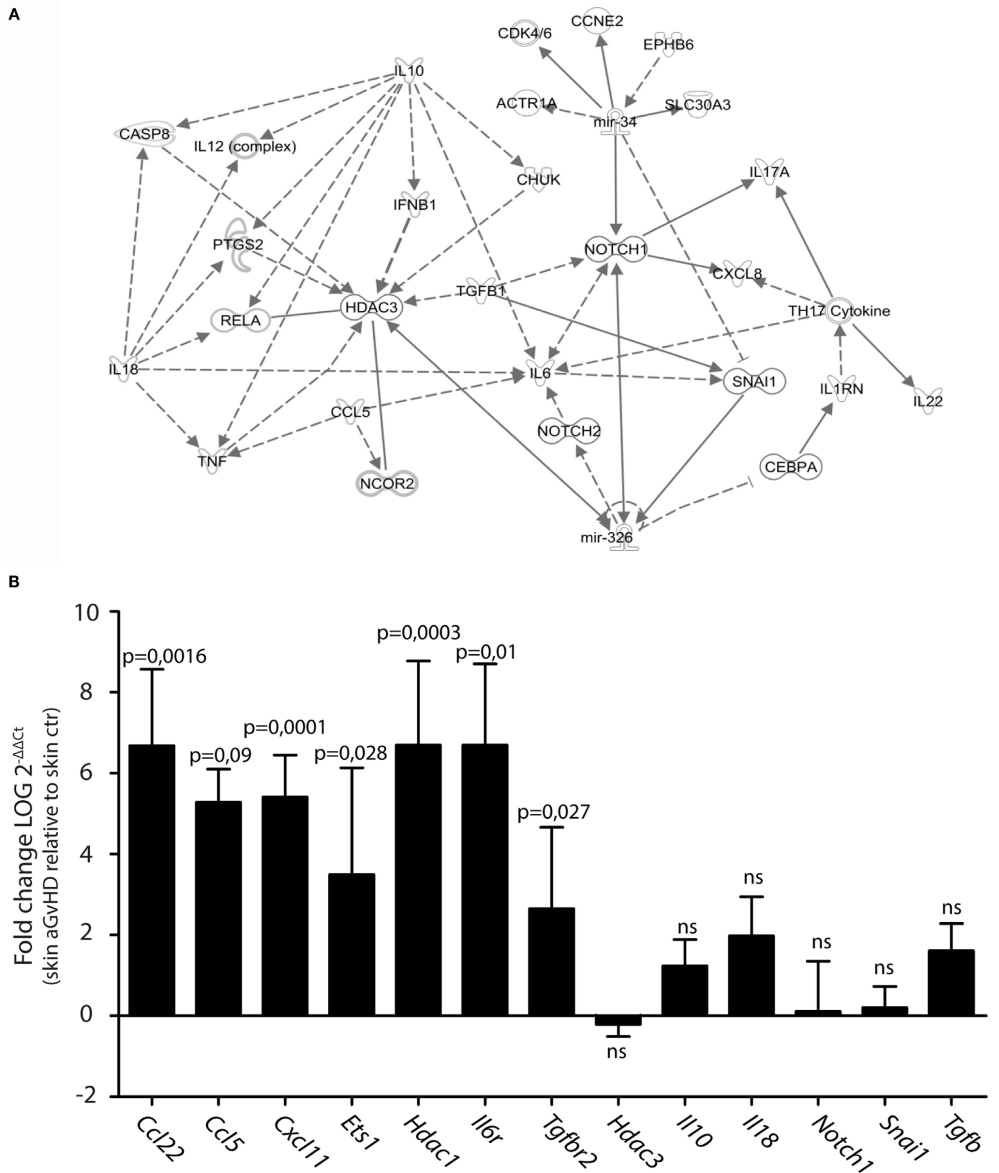
**Prediction of mRNA targets by Ingenuity Pathway Analysis**. **(A)** A network of possible mRNA targets and affected biological pathways for miR-34b and miR-326 was constructed using QIAGEN’s IPA software. Dashed lines represent indirect interactions, while solid lines indicate direct interactions. **(B)** mRNA expression analysis by qRT-PCR analysis in skin samples from controls (*n* = 6) or aGvHD rats (*n* = 6). Samples were normalized to an endogenous control (*B2m*) and expression levels reported as log of 2^−ΔΔCt^ ± SD. Significance was calculated using the Student’s *t*-test (two-tailed) with Welch’s correction.

We searched for predicted gene targets for miR-326 and miR-34b using QIAGEN’s IPA software. We filtered for genes involved in T cell activation and GvHD responses to narrow down the list of predicted targets. To assess how these predicted targets could interact, we performed an IPA pathway analysis. This analysis demonstrated that both miR-326 and miR-34b may converge on at least two gene targets, *Notch1* and *Snai1* (Figure 2A), although these genes were not differentially expressed in skin tissues from aGvHD rats compared to controls (Figure 2B). miR-326 may additionally regulate the histone deacetylase 3 (*Hdac3*) gene. While *Hdac3* was not differentially expressed, the closely related *Hdac1* gene was over-expressed in skin tissues from aGvHD rats compared to controls (Figure 2B). In addition, several cytokines and cytokine receptors were indirectly linked to these miRNAs and their potential primary targets. Quantitative RT-PCR expression analyses showed significant upregulation in aGvHD rats of *Ccl5, Ccl22, Cxcl11, Il6r*, and *Tgfbr2*, and a trend toward upregulation of *Il10, Il18*, and *Tgfb* (Figure 2B). In addition, we detected upregulation of another predicted target gene for miR-326, the transcription factor *Ets1* (Figure 2B).

### miRNA Expression Profiling in the Lung

**Figure 3:**
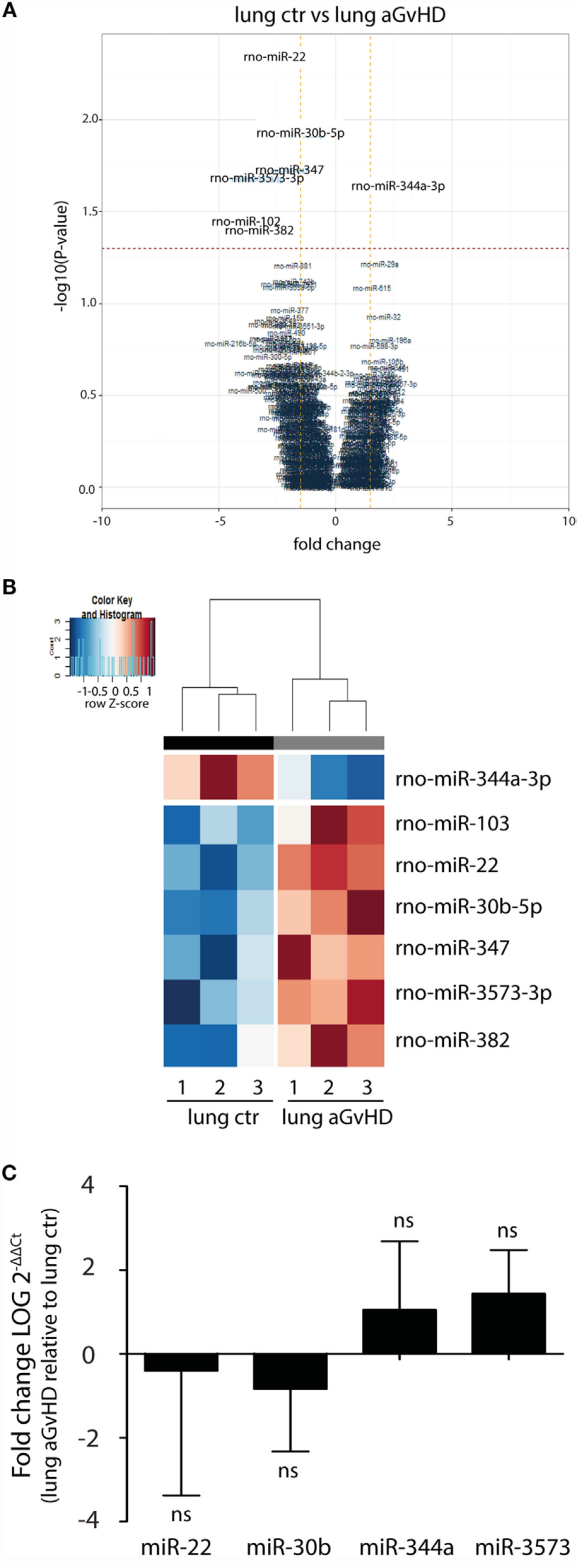
**miRNA expression signature in lung during aGvHD**. **(A)** Volcano plot showing the relationship between fold change and significance between the two groups. The horizontal dashed line indicates cutoff for significance *p* < 0.05 (−log10 *p*-value >1.3) and the vertical lines for fold change ≥1.5/≤−1.5. **(B)** Heatmap showing unsupervised hierarchical clustering of differentially expressed miRNAs in lung between control (*n* = 3) and aGvHD (*n* = 3) animals. Each column represents an individual rat. Relative fold change is indicated by the color scale (red: high; blue: low). **(C)** qRT-PCR analysis of miRNA expression in lung of control (*n* = 6) or aGvHD (*n* = 6) rats. Samples were normalized to endogenous controls and expression levels reported as log of 2^−ΔΔCt^ ± SD. Significance was calculated using the Student’s *t*-test (two-tailed) with Welch’s correction.

In our rat model of aGvHD, the lungs appear macroscopically normal during aGvHD. However, we have previously detected increased expression of IFN-γ in the lungs during aGvHD, suggesting that lungs are affected^3^. miRNA expression profiling of lung tissues demonstrated differential expression of seven miRNAs, with downregulation of miR-344a-3p (−2.36-fold change) and upregulation of miR-103 (4.04-fold change), miR-22 (2.72-fold change), miR-30b-5p (1.51-fold change), miR-347 (1.95-fold change), miR-382 (2.82-fold change), and miR-3573-3p (3.32-fold change) (Figures 3A,B). Quantitative RT-PCR validation of three upregulated miRNAs with low *p*-values and the one downregulated miRNA did not confirm the differential expression in aGvHD samples over controls (Figure 3C). The mild pathology observed in lung may imply that putative differences between aGvHD and control rats may be too low to be detected regularly.

### Differential Expression of miR-146a and miR-155 in Skin

**Figure 4:**
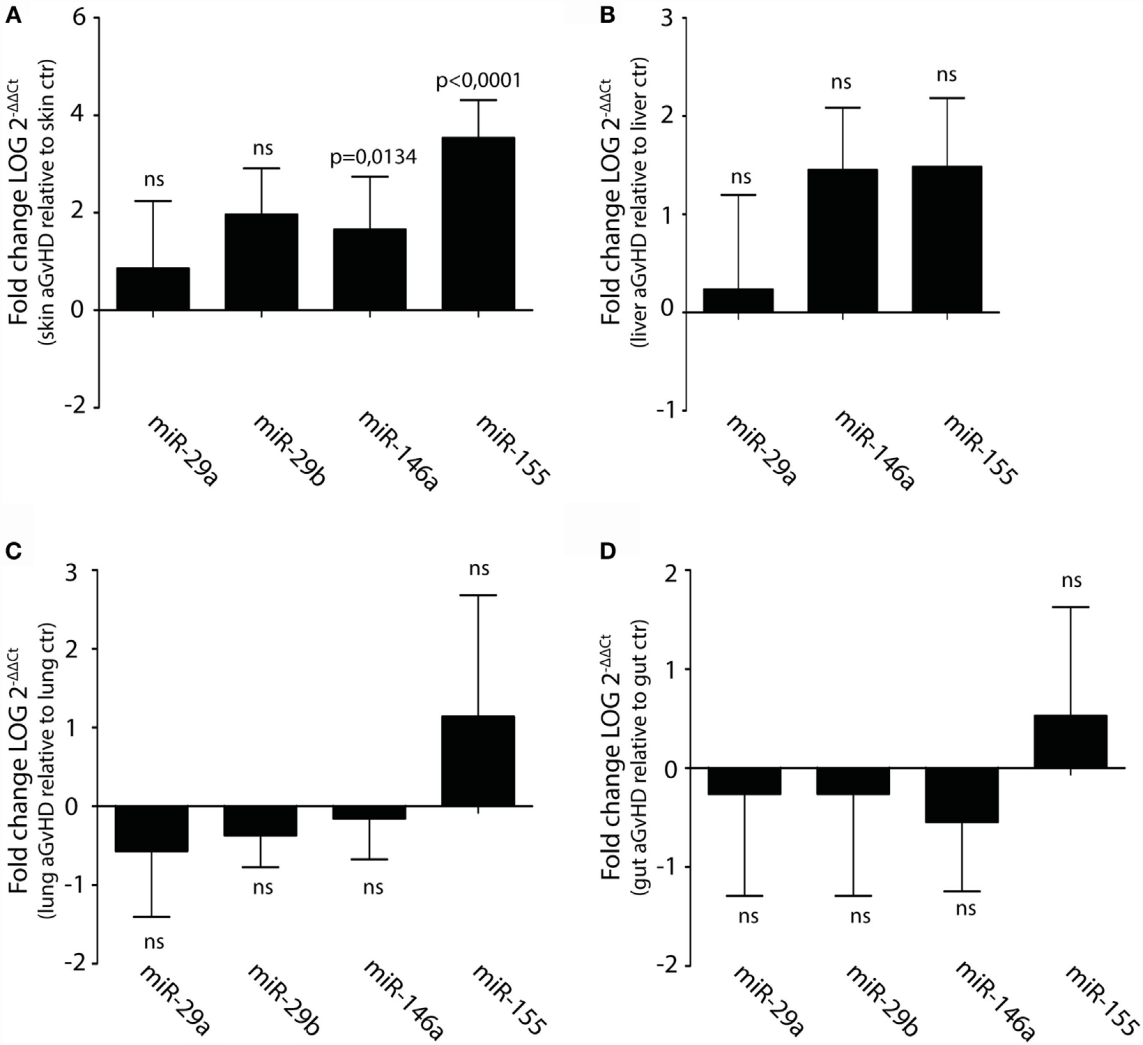
**Upregulation of miR-146a and miR-155 in skin but not liver, lung, and intestines during aGvHD**. qRT-PCR analysis of miRNA expression in **(A)** skin (controls, *n* = 6; aGvHD, *n* = 6), **(B)** liver (controls, *n* = 6; aGvHD, *n* = 6), **(C)** lung (controls, *n* = 6; aGvHD, *n* = 6), or **(D)** intestines (controls, *n* = 6; aGvHD, *n* = 6). Samples were normalized to endogenous controls and expression levels reported as log of 2^−ΔΔCt^ ± SD. Significance was calculated using the Student’s *t*-test (two-tailed) with Welch’s correction.

Upregulation or downregulation of miRNAs previously implicated in aGvHD pathogenesis such as the miR-17-92 cluster, miR-29a, miR-29b, miR-146a, or miR-155 did not reach significance in the NanoString analysis. However, several of these miRNAs were detected as differentially expressed below the threshold cutoff of *p* < 0.05 (data not shown). We therefore tested their expression in tissues by qRT-PCR. In this analysis, we also included liver, which is another target tissue for aGvHD. We found clear upregulation of miR-146a and miR-155, but not of miR-29a, miR-29b, miR-19b, or miR-20a in skin (Figure 4A and data not shown). None of the tested miRNAs were significantly differentially expressed in liver (Figure 4B), lung (Figure 4C), or gut tissues (Figure 4D).

### The miRNA Expression Pattern of Intestinal T Cells Is More Similar to Blood T Cells than to Intestinal Tissue

**Figure 5:**
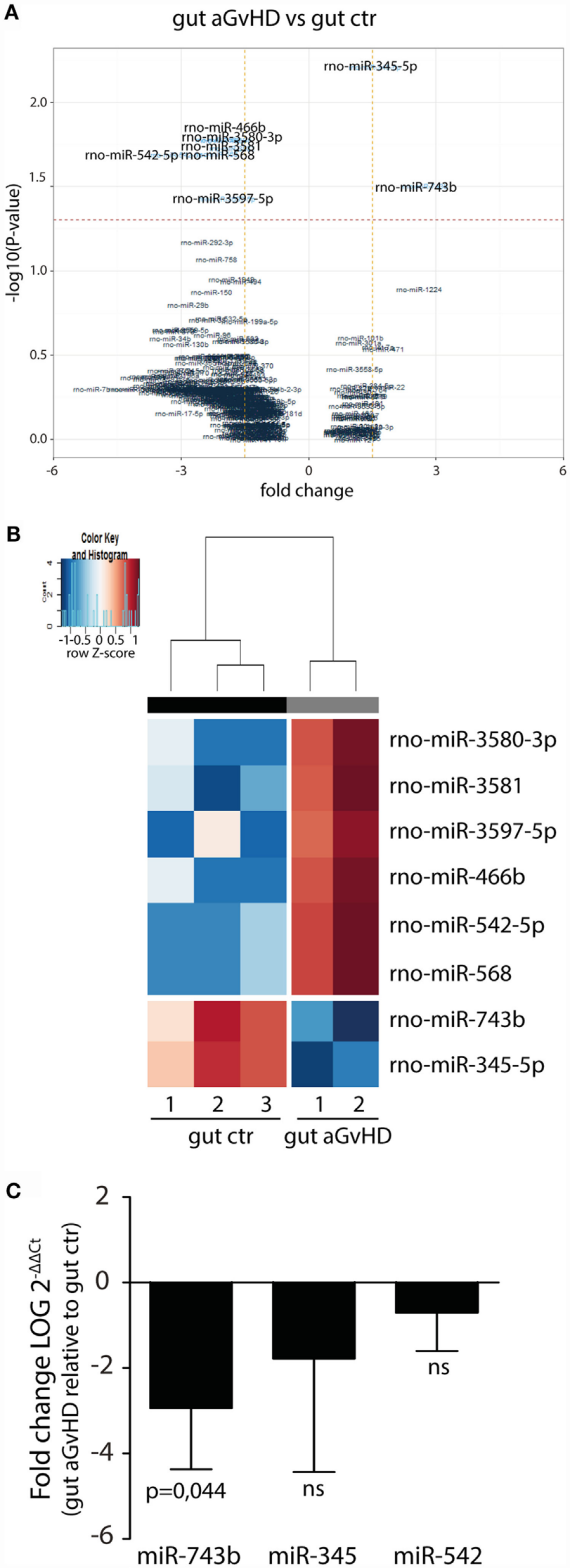
**miRNA expression signature in small intestines during aGvHD**. **(A)** Volcano plot showing the relationship between fold change and significance between the two groups. The horizontal dashed line indicates cutoff for significance *p* < 0.05 (−log10 *p*-value >1.3) and the vertical lines for fold change ≥1.5/≤−1.5. **(B)** Heatmap showing unsupervised hierarchical clustering of differentially expressed miRNAs in gut between control (*n* = 3) and aGvHD (*n* = 2) animals. Each column represents an individual rat. Relative fold change is indicated by the color scale (red: high; blue: low). **(C)** qRT-PCR analysis of miRNA expression in small intestines of control (*n* = 6) or aGvHD (*n* = 6) rats. Samples were normalized to endogenous controls and expression levels reported as log of 2^−ΔΔCt^ ± SD. Significance was calculated using the Student’s *t*-test (two-tailed) with Welch’s correction.

In the small intestine, eight miRNAs were differentially expressed in aGvHD rats compared to controls. Specifically, upregulation of miR-466b (1.99-fold change, *p* = 0.017), miR-542-5p (3.09-fold change, *p* = 0.020), miR-568 (2.14-fold change, *p* = 0.020), miR-3580-3p (1.99-fold change, *p* = 0.017), miR-3581 (1.79-fold change, *p* = 0.019), and miR-3597-5p (1.9-fold change, *p* = 0.037), and downregulation of miR-345-5p (−1.56-fold change, *p* = 0.006) and miR-743b (−2.74-fold change, *p* = 0.031) were detected for aGvHD samples compared to controls (Figures 5A,B). Quantitative RT-PCR validation of samples obtained from the same and another independent transplantation experiment confirmed downregulation of miR-743b (Figure 5C). There was also a trend toward downregulation of miR-345-5p (*p* = 0.140), although not to significance (Figure 5C). The upregulation of miR-542-5p or miR-466b was not confirmed in the validation samples (Figure 5C and data not shown).

**Figure 6:**
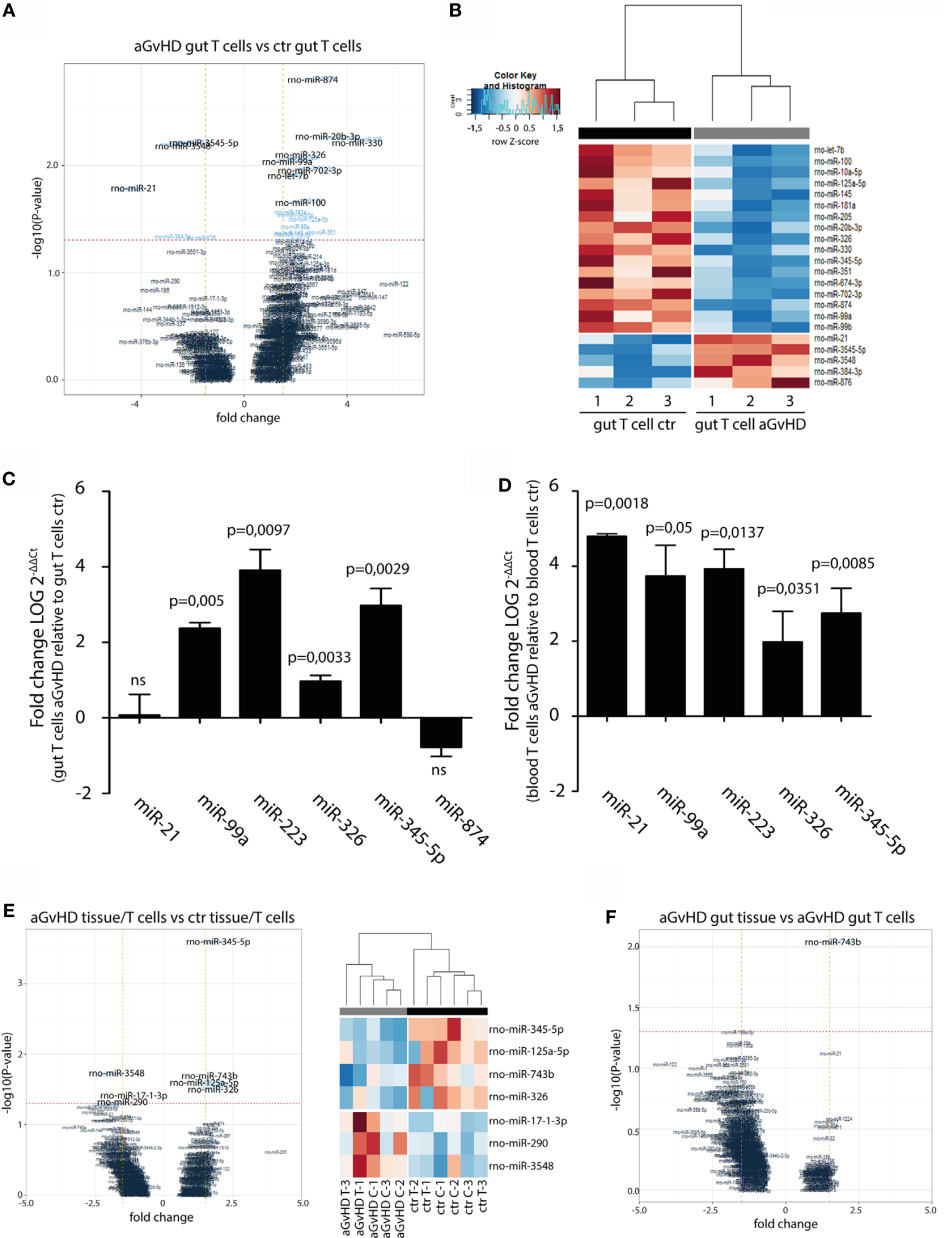
**miRNA expression in intestinal T cells and comparison to intestinal tissues**. **(A)** Volcano plot showing the relationship between fold change and significance between the two groups. The horizontal dashed line indicates cutoff for significance *p* < 0.05 (−log10 *p*-value >1.3) and the vertical lines for fold change ≥1.5/≤−1.5. **(B)** Heatmap showing unsupervised hierarchical clustering of differentially expressed miRNAs in intestinal T cells between control (*n* = 3) and aGvHD (*n* = 3) animals. Each column represents an individual rat. Relative fold change is indicated by the color scale (red: high; blue: low). **(C)** qRT-PCR analysis of miRNA expression in intestinal T cells from control (*n* = 3) or aGvHD (*n* = 3) rats. Samples were normalized to endogenous controls and expression levels reported as log of 2^−ΔΔCt^ ± SD. Significance was calculated using the Student’s *t*-test (two-tailed) with Welch’s correction. **(D)** qRT-PCR analysis of miRNA expression in peripheral blood T cells from control (*n* = 3) or aGvHD (*n* = 3) rats. Samples were normalized to endogenous controls and expression levels reported as log of 2^−ΔΔCt^ ± SD. Significance was calculated using the Student’s *t*-test (two-tailed) with Welch’s correction. **(E)** Volcano plot (left panel) and heatmap (right panel) showing differentially expressed miRNAs in intestinal T cells (C) and intestinal tissue (T) from aGvHD rats compared to intestinal T cells (C) and intestinal tissue (T) from controls. **(F)** Volcano plot showing the relationship between fold change and significance between the intestinal tissue and intestinal T cells from aGvHD rats. The horizontal dashed line indicates cutoff for significance *p* < 0.05 (−log10 *p*-value >1.3) and the vertical lines for fold change ≥1.5/≤−1.5.

In order to specifically address changes in miRNA profiles in T cells from target tissues, we profiled miRNA expression in TCRαβ^+^ T cells purified from the IEL compartment. Although aGvHD in this rat model likely affects the skin more than any other tissue, we chose to purify T cells from the intestines due to the technical difficulty in purifying high enough T cell numbers from the skin. Total RNA from intestinal T cells was subjected to NanoString analysis as above with samples obtained from rats suffering from aGvHD (*n* = 3) or from control rats (*n* = 3). Here, 17 miRNAs were downregulated and 5 miRNAs were upregulated (fold change >1.3, *p* < 0.05, Table 2) in animals with aGvHD compared to controls (Figures 6A,B). In particular, downregulation of miR-874 (−2.89-fold change, *p* = 0.002), mir-20b (−3.37-fold change, *p* = 0.005), miR-330 (−4.11-fold change, *p* = 0.006), and miR-326 (−2.22-fold change, *p* = 0.008), and upregulation of miR-3545-5p (1.97-fold change, *p* = 0.006), miR-3548 (2.59-fold change, *p* = 0.006), and miR-21 (3.52-fold change, *p* = 0.017) was observed. Validation by qRT-PCR analysis of miR-874 from another independent experiment showed a trend toward downregulation in intestinal T cells, while miR-21 was not differentially expressed (Figure 6C). Moreover, we found that miR-99a, miR-326, and miR-345-5p, which were all indicated as downregulated by NanoString were detected as upregulated by qRT-PCR (Figure 6C). We also observed upregulation of miR-223 in intestinal T cells (Figure 6C). When compared to T cells from peripheral blood, qRT-PCR analyses of purified TCRαβ^+^ blood T cells from the same rats indicated a similar expression pattern, with upregulation of miR-21, miR-99a, miR-223, miR-326, and miR-345-5p (Figure 6D), indicating that the GvHD grade during sampling of T cells may be crucial in terms of miRNA expression. We next sought to identify miRNAs commonly dysregulated in both intestinal tissue and purified intestinal T cells, based on the data obtained from NanoString analysis. Here, miR-125a-5p, miR-326, miR-345-5p, and miR-743b were downregulated in both intestinal tissue and intestinal T cells, while miR-17-1-3p, miR-290, and miR-3548 were upregulated (Figure 6E). Lastly, a comparison of intestinal tissue with intestinal T cells from aGvHD rats showed that only miR-743b were significantly differentially expressed (Figure 6F).

**Table 2:** **Differentially expressed miRNA in TCRαβ^+^ T cells from the IEL compartment**.

Gene	Accession ID	Fold change	*p*-value
Rno-miR-874	MIMAT0005284	−2.89	0.002
Rno-miR-20b-3p	MIMAT0003212	−3.37	0.005
Rno-miR-330	MIMAT0004641	−4.11	0.006
Rno-miR-3545-5p	MIMAT0017799	1.97	0.006
Rno-miR-3548	MIMAT0017806	2.59	0.006
Rno-miR-326	MIMAT0000560	−2.22	0.008
Rno-miR-99b	MIMAT0000821	−1.85	0.009
Rno-miR-702-3p	MIMAT0017885	−2.75	0.011
Rno-let-7b	MIMAT0000775	−1.95	0.012
Rno-miR-21	MIMAT0000790	3.52	0.017
Rno-miR-100	MIMAT0000822	−2.47	0.021
Rno-miR-181a	MIMAT0000858	−1.80	0.027
Rno-miR-345-5p	MIMAT0000594	−1.99	0.029
Rno-miR-125a-5p	MIMAT0000829	−2.52	0.031
Rno-miR-99a	MIMAT0000820	−1.96	0.037
Rno-miR-205	MIMAT0000878	−9.01	0.040
Rno-miR-351	MIMAT0000608	−2.92	0.042
Rno-miR-145	MIMAT0000851	−1.69	0.042
Rno-miR-10a-5p	MIMAT0000782	−1.87	0.043
Rno-miR-384-3p	MIMAT0005310	2.44	0.045
Rno-miR-876	MIMAT0012843	1.67	0.047
Rno-miR-674-3p	MIMAT0005330	−1.98	0.049

**Figure 7:**
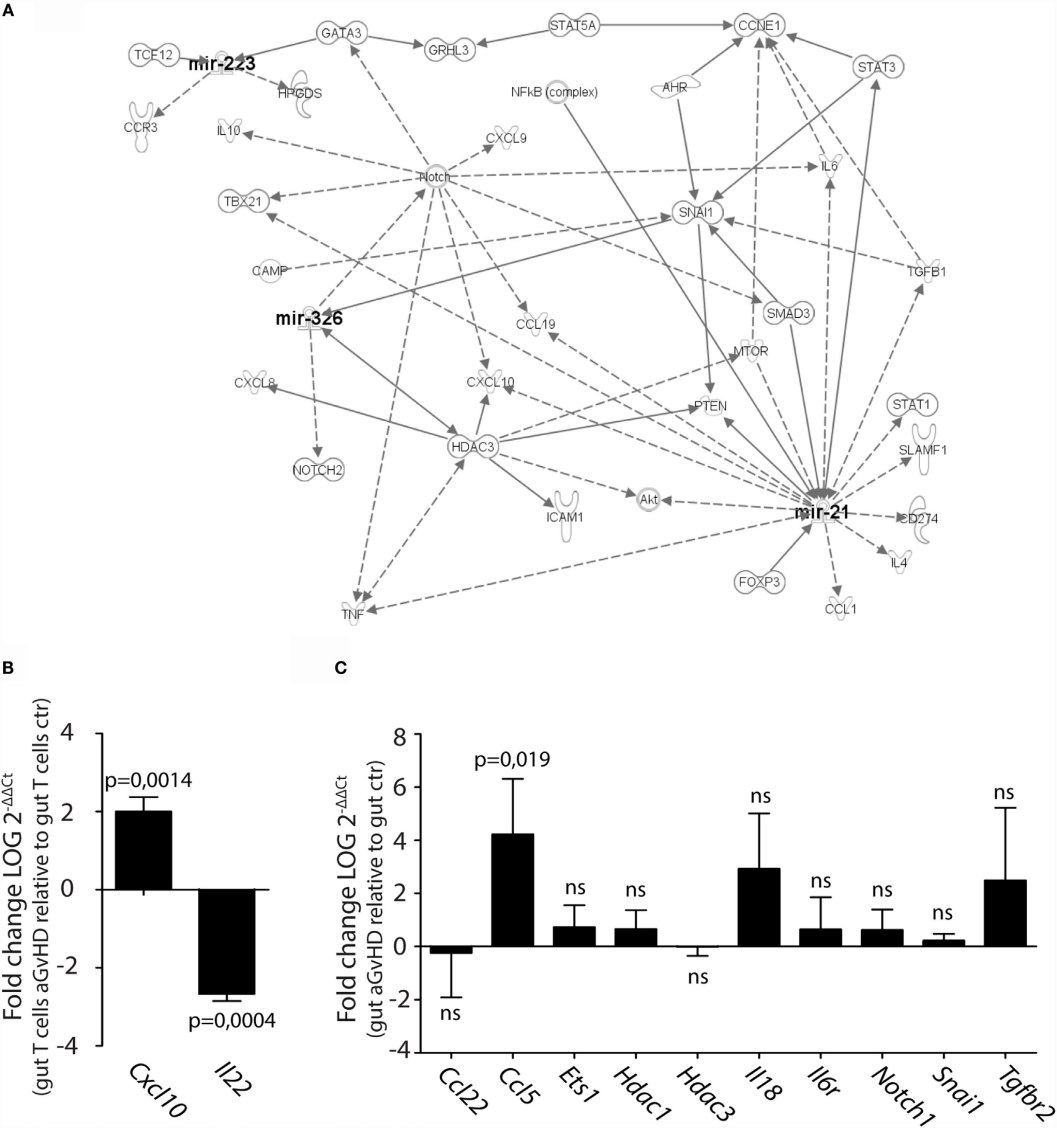
**IPA network analysis of differentially expressed miRNAs in intestinal tissue and T cells**. **(A)** A network of possible mRNA targets and affected biological pathways for miR-21, miR-223, and miR326 was constructed using QIAGEN’s IPA software. Dashed lines represent indirect interactions, while solid lines indicate direct interactions. **(B,C)** mRNA expression analysis by qRT-PCR analysis of **(B)** intestinal T cells from controls (*n* = 3) or aGvHD rats (*n* = 3) or **(C)** intestinal tissue from controls (*n* = 6) or aGvHD rats (*n* = 6). Samples were normalized to endogenous control (*B2m*) and expression levels reported as log of 2^−ΔΔCt^ ± SD. Significance was calculated using the Student’s *t*-test (two-tailed) with Welch’s correction.

We next performed pathway analysis of the miRNAs found significantly upregulated by qPCR analysis in intestinal tissue and/or intestinal T cells in order to analyze whether and how these miRNAs interact with each other. As input for the analysis, we chose miR-21, miR-99a, miR-223, miR-326, miR-345-5p, and miR-743b, and also genes involved in GvHD pathogenesis as well as T cell activation, proliferation, and migration. The generated network depicted in Figure 7A demonstrates that miR-21, miR-223, and miR-326 may all interact in the same network of molecular responses related to T cell activation and migration. miR-99a, miR-345-5p, or miR-743b was not found associated with any of the other miRNAs or to genes related to T cell activation. qPCR expression analysis of intestinal T cells showed upregulation of the inflammatory chemokine *Cxcl10*, identified in the network, and downregulation of *Il22* expression (Figure 7B). In intestinal tissues, increased expression of *Ccl5* and a tendency toward increased expression of *Il18* was detected in aGvHD rats, similarly to the skin. As for the skin, major hubs represented by *Notch1, Hdac3*, and *Snai1* were identified, but none of these genes were found to be differentially expressed in intestinal tissues from aGvHD rats (Figure 7C).

## Discussion

MicroRNAs are now considered central regulators of gene expression, and miRNA expression is tightly linked to different cellular responses and may be predictive of underlying molecular mechanisms in complex diseases such as aGvHD. Using a rat model of aGvHD, we comprehensively analyzed and compared the miRNA expression profiles in aGvHD target tissues and in T cells isolated from intestines or blood. As miRNA may be variably expressed in distinct tissues, and between species, the contribution from our rat model may be important for evaluating miRNAs that are central regulators of GvHD pathology. We also identified genes that may be targeted by one or more of the miRNAs differentially expressed during aGvHD in the rat, although these results does not make firm inferences. Further direct functional tests such as luciferase assays are required to identify the interaction between a specific miRNA and its target gene.

Although alloreactive T cells infiltrate target tissues and are the direct cause of tissue destruction, there was little overlap of differentially expressed miRNAs in skin, intestinal, and lung tissues as a consequence of aGvHD. Macroscopically, the skin and the oral mucosae are the most affected tissues in our rat model of aGvHD, with clear bruising and lesions. While the intestines, lung, and liver appear macroscopically less affected, we observe infiltration of donor T cells to the intestines and upregulation of IFN-γ in all tissues indicating that these tissues are targeted by the disease (Boieri et al., unpublished observation). In the skin, NanoString analysis indicated differential expression of miR-34b and miR-326 in rats with aGvHD compared to controls, although only a trend was observed by qRT-PCR. The miR-34 family was recently shown to be upregulated in the gut of patients during severe aGvHD (18). We did not detect differential expression of miR-34b or miR-326 in intestinal tissues of rats, and this may reflect the more severe pathology that is observed in skin rather than intestines of rats but also differences in tissue pathology. Rather in intestines, we detected significant downregulation of miR-743b in rats with aGvHD disease and a trend toward decreased expression of miR-345-5p. MiR-743b has not previously been associated with aGvHD or with any immune-related functions and thus represents an interesting miRNA for further investigations into its role in aGvHD pathology. Also, miR-326 has not previously been implicated in aGvHD in any species. It has, however, been implicated in immune-related disorders and is upregulated during the relapsing phase of multiple sclerosis, possibly contributing to T helper (Th) 17 differentiation (19, 20). We also detected differential expression of miR-326 in intestinal and blood T cells but not in intestinal tissues. As we were unable to purify high enough numbers of skin T cells, it is presently unknown whether the differentially expressed miR-326 stems from the infiltrating alloreactive T cells or from other immune or non-immune cells in the skin tissue.

Ingenuity Pathway Analysis, which incorporates existing data on genes regulated by miRNAs from the literature, indicated that both miR-34b and miR-326 may share at least two target genes, *Notch1* and *Snai1* (21–24). Snail proteins are zinc-finger transcription factors classically associated with epithelia to mesenchymal transition (25). However, Snail proteins may also regulate immune responses, and a recent study indicated that Snail-expressing keratinocytes may induce production of proinflammatory cytokines in response to TGF-β (26). This could correlate with the observed increased inflammatory cytokine expression levels in the skin, although *Snai1* was not differentially expressed in our analysis. Target prediction and pathway analysis identified *Notch1* as a possible target in the network of differentially expressed miRNAs and genes in both skin and intestinal T cells, where it is potentially targeted by both miR-21 and miR-326. Apart from its role in T cell development in the thymus, Notch1-dependent signaling in peripheral T cells may lead to increased production of inflammatory cytokines (27). Its role in regulating peripheral T cell activation and in driving differentiation of T helper subsets is still incompletely defined and likely context dependent. Notch1 signaling is suggested to contribute to both generation of Tregs (28) and Th1 cells (29). We have previously observed increased frequencies of Tregs in skin of rats with severe aGvHD (Boieri et al., unpublished observation). In gliomas, miR-326 has been shown to participate in a feedback loop with Notch, where miR-326 expression is specifically repressed by Notch (22). We could not detect differential expression of *Notch1* or *Snail* in rats with aGvHD. However, this might indicate that the mRNA expression of these genes is counter regulated to normal levels by miRNAs. This pathway could certainly be interesting to study in more detail in the future.

The miR-17-92 cluster, miR-146a, and miR-155 have been previously implicated in aGvHD, where they are shown to regulate different aspects of T cell allo-activation (4, 10, 12, 13, 30) or T helper cell differentiation (31). These miRNAs were not among the significantly differentially expressed miRNAs as detected by the NanoString analyses, but we clearly observed higher expression levels of miR-146a and miR-155 in skin tissue, but not in intestines, liver, and lung, by qRT-PCR. Both miR-17 and miR-19, as representatives of the miR-17-92 cluster, were tested in all tissues, but no changes were observed between controls and aGvHD rats (data not shown). The expression of miR-146a and miR-155 is induced by lipopolysaccharide, which in the context of aGvHD is released as a consequence of breached epithelial barriers during pretransplantation irradiation. miR-155 is involved in a number of inflammatory responses, and its expression is induced by inflammatory cytokines. Importantly, miR-155 is shown to repress *Socs1* expression leading to increased proinflammatory cytokine production (32), which could correlate with the higher expression levels of inflammatory cytokines in skin observed in this study. miR-146a is shown to target *Traf6* and *Irak* and thereby negatively regulate cytokine signaling and activation of the transcription factor NF-κB (33). miR-146a is also suggested to regulate differentiation of Th1 cells and more recently to critically control Treg functions through direct targeting of STAT1 (14).

We obtained discrepant results on the expression of miRNAs in intestinal T cells when comparing NanoString results with qRT-PCR analysis. Although the same miRNAs were detected as differentially expressed, miRNAs observed as downregulated by NanoString were upregulated in cells when validated by qRT-PCR. Although great care was taken in sampling cells and tissues at the same severe stage of the disease, we cannot exclude differences in the stage of the disease at the time of sampling. We have previously observed that the composition of intestinal T cells may vary from rat to rat within the same strain and also that the T cell responses in the gut may vary between experiments and between rats within the same experiment. In contrast, T cell markers and responses are stable in lymphatic tissues and in the skin (Boieri et al., unpublished observation). It could be that initially downregulated miRNAs are upregulated as a compensatory feedback mechanism at late-stage aGvHD responses or *vice versa*. Interestingly, we detected reduced expression of *Il22* in T cells from the intestine. IL-22 is shown to contribute to the severity of aGvHD pathogenesis, being produced by both innate and adaptive immune cells present in intestinal or skin tissues (34, 35). The observed reduction in *Il22* expression in T cells isolated from rats with severe aGvHD could potentially represent a response to negative feedback mechanisms operating at the height of the proinflammatory responses. Whether the *Il22* gene is regulated by a specific miRNA in the intestines of rats with aGvHD disease is currently unknown.

It appears from our data that tissue-specific expression patterns of miRNAs exist, with little overlap between tissues. This may be surprising, due to the fact that these tissues are infiltrated by alloreactive donor T cells during aGvHD. However, resident cells in tissues are affected by the disease and also other immune cells are recruited to the inflamed tissues. Our analysis encompassed the whole tissue, and no distinctions were made on the contributions from different cell types, immune or non-immune. It could be that the majority of dysregulated miRNA expression patterns in tissues are due to infiltrating T cells, but the unique cellular composition of different tissues likely also affects the total miRNA expression patterns. In this respect, further dissection of miRNA expression in distinct cell populations within skin or intestines could be informative. It therefore appears that profiling the miRNA expression pattern of peripheral blood T cells may not necessarily predict miRNA expression patterns in tissues, although possibly reflecting the miRNA profile of tissue-infiltrating T cells.

In conclusion, we show that the miRNA repertoire is changed in tissues and in T cells during aGvHD in the rat, and that there are wide variations in the miRNA expressed in skin, lung, or intestines. We also found that there was little overlap between miRNA expressed in intestinal T cells with those of intestinal tissues, indicating tissue-specific changes in miRNA in response to the damage evoked by T cells in aGvHD. We did, however, find that the differentially expressed miRNA in skin or in intestinal T cells could be part of a network of genes and miRNA involved in the regulation of T cell activation. A better understanding of the complex regulation of miRNAs and their target genes during aGvHD may enhance our understanding of the disease and possibly aid in the development of better treatment strategies to ameliorate the severity of aGvHD.

## Author Contributions

AD, JN, RC, DJ, and MI conceived and designed the study; DJ, MB, IB, and PS acquired data; DJ, RC, MB, IB, MI, JN, AD, PS, and RD analyzed and interpreted data; MI drafted the manuscript; DJ, RC, and MB revised the manuscript; and DJ, MB, RC, JN, IB, AD, MI, PS, and RD approved the final version of the manuscript.

## Conflict of Interest Statement

The authors declare that the research was conducted in the absence of any commercial or financial relationships that could be construed as a potential conflict of interest.

## Footnotes

^1^www.moma.dk/normfinder-software

^2^www.bioconductor.org

^3^Boieri et al. (unpublished observation).

## Abbreviations

aGvHD, acute graft-versus-host disease; APC, antigen-presenting cells; IELs, intraepithelial lymphocytes; IPA, Ingenuity Pathway Analysis; TGF-β, transforming growth factor beta; Th, T helper; Treg, T regulatory cells.
