# Supplementary material for: Tissue-Specific Expression Patterns of MicroRNA during Acute Graft-versus-Host Disease in the Rat
Source: Front Immunol. 2016 Sep 16;7:361. doi: 10.3389/fimmu.2016.00361 (PMC5025478; doi:10.3389/fimmu.2016.00361)
Supplement: Supplementary file 1 [file Image_1.PDF]

Supplementary Figure 1

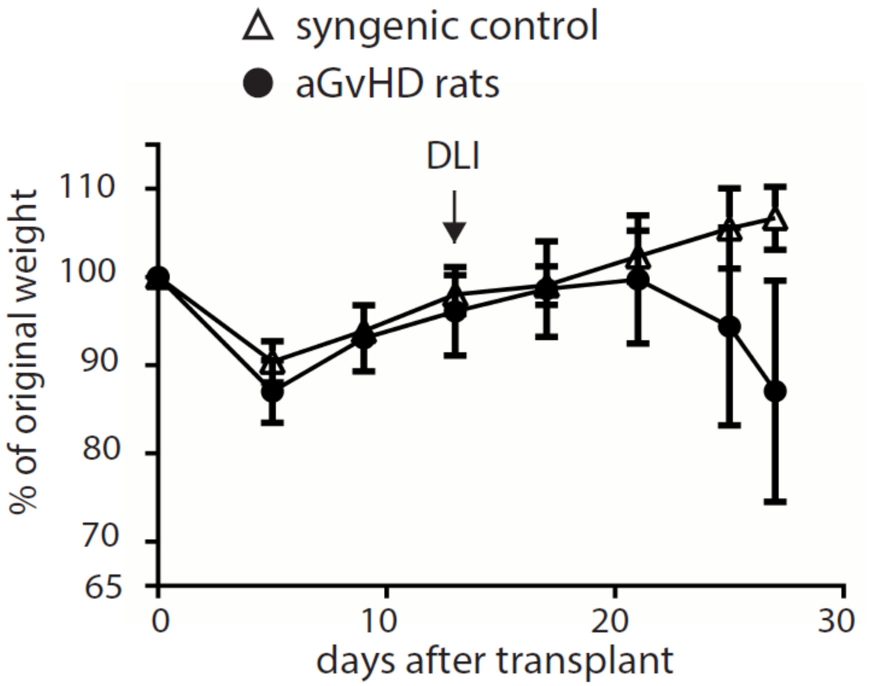

Monitoring of weight in transplanted rats before and after DLI (Δ: syngeneic transplantations (PVG.7B to PVG); ●: allogeneic transplantations (PVG.7B to PVG.1N).
